# Maintaining disorder: the micropolitics of drugs policy in Iran

**DOI:** 10.1080/01436597.2017.1350818

**Published:** 2017-08-09

**Authors:** Maziyar Ghiabi

**Affiliations:** a Department of Politics and International Relations, University of Oxford, Oxford, UK

**Keywords:** Iran, ethnography of the state, addiction, civil society, drugs, micropolitics

## Abstract

This article analyses the ways in which the state ‘treats’ addiction among precarious drug (ab)users in Iran. While most Muslim-majority as well as some Western states have been reluctant to adopt harm reduction measures, the Islamic Republic of Iran has done so on a nationwide scale and through a sophisticated system of welfare intervention. Additionally, it has introduced devices of management of ‘addiction’ (the ‘camps’) that defy statist modes of punishment and private violence. What legal and ethical framework has this new situation engendered? And what does this new situation tell us about the governmentality of the state? Through a combination of historical analysis and ethnographic fieldwork, the article analyses the paradigm of government of the Iranian state with regard to disorder as embodied by the lives of poor drug (ab)users.

‘I call the camps if someone calls me!’

Police Officer in Arak, September 2014.

I have the feeling we are crying on a grave which is empty.^1^ How many people, arrested for drug addiction and sent to compulsory camps, have actually been in front of a judge? And, if this has happened, had the judge said anything to them about treatment? I doubt that we can find ten people in the whole country who have met a judge before going to a camp, so I think the question here is something else and it is not related to compulsory treatment. … The problem, it seems to me, is that the question is not medical and therapeutic, but one of social and political control.

Professor Emran Razzaghi, International Addiction Studies Conference, Tehran, 10 September 2014.

## Introduction: ethnography of a policy

In the southern district of Tehran’s Bazaar, between Mowlavi Street and Shoosh Street, there are four public gardens. The biggest and most popular of these is Harandi Park, which stands at the heart of the old neighbourhood of Darvazeh Ghar. Since 2014, Harandi Park and, to a similar extent, the others have been *lieux* of encounter of large groups of drug users who camp there with tents, sleeping bags, bonfires and piles of cardboard on the ground. Over the warm seasons – between March and November – the number of street drug users residing within the perimeter of the parks and the connecting alleys reaches three to four thousand, with additional visitors towards the evening spleen.^2^

While on a late-morning stroll across the lawn, I encountered waste collectors and gardeners working their way between groups of drug users, chatting or just passing through their circles. Every now and then, a police motorbike would ride on the main road circumscribing the park or in the middle of it, with not much preoccupation with the people smoking heroin or *shisheh*, the word for methamphetamine (crystal meth) in Iran. The entrance of a bigger tent, close to a smelly empty pool that served as an open-air loo, was animated by the bustling of a dozen people. I was told later that the tent is where the main distribution of *gart* (heroin) and *shisheh* in the Harandi area takes place and that it is the centre of gravity of the park. This is not an underground, hidden site of criminality or an unseen zone of crisis/disorder; the park stands in the middle of one of Tehran’s most popular neighbourhoods, which has a symbiotic relation to its great bazaar and is located close to the main metro line (Line 1) connecting the wealthy north with the city’s poorer southern districts. In contrast to the ever-lasting declarations of the ‘War on Drugs’ and the ever-increasing number of drug arrests, the situation in Harandi casts light on a different approach based on limited tolerance of public drug use and the tacit acceptance of street hustling, more or less.

Activism among civil society groups and non-governmental organisations (NGOs) has attracted public attention to this place, which by 2015 had become a leitmotif of debate around drugs policy in Tehran. The city municipality and the mayor of the district denied their acceptance of the situation and reiterated that there is no plan to transform Harandi into a social experiment of de facto drug decriminalisation.^3^ A temporary alternative to the plans for collection of drug (ab)users, it refrains from the incarceration or forced treatment of the compulsory camps, which I will discuss in detail in this article. Instead, by having large gatherings of so-called ‘risky’ drug (ab)users concentrated in specific areas such as Harandi Park, social workers and medical personnel can intervene with harm reduction services (eg needle exchange, condom distribution, etc.) and attempt to introduce them into the cycle of treatment, notably methadone maintenance, even though many of these drug (ab)users are meth smokers or polydrug users, for whom methadone, a pharmacological opiate substitute, is not effective. The ‘dispersion of risk’ is reduced, according to public officials, who imply that without Harandi the whole of Tehran would be a scene of open-air drug use and drug hustling, with the spectre of HIV epidemics looming all too large over the populace. It would be *uncontrollable*.

This ethnographic vignette casts light on the routines and connections of everyday drugs policy in Iran. This article, similarly, analyses the micropolitics of ‘addiction’ policies and the way Iranians and the Iranian state treat drug (ab)users. (Ab)user, here, suggests the ambiguity that rules the definition of ‘addiction’ as a social artefact and the addict as its primary actor. The limit between using and abusing drugs, indeed, rests ultimately upon a judgement on the status of the addict; it is the outcome of legal prosecution, economic degradation and policing practices. While most Muslim-majority as well as some Western states have been reluctant to adopt welfare-oriented measures (eg harm reduction) towards drug users, the Islamic Republic of Iran has done so on a nationwide scale enshrined in the 2010 drug law reform. The article asks: What legal and ethical framework has this new situation engendered? And what does this tell us about state practices with regard to zones of social disorder and crisis? The article does question whether Iran is a theocracy, a republic or just another authoritarian state as it has been the subject of endless scholarly work. The article is not interested in ‘what is the nature of power’. Rather I discuss, following Deleuze's incitement, ‘in what ways power is exercised, in what place it is formed and why it is everywhere’.^4^ In this way the article shows how power works in the micropolitical dimension and how it defies top-down expositions of politics in the Islamic Republic.

Based on ethnographic research (immersion in the field) and more than 50 semi-structured interviews carried out between 2012 and 2016, the article analyses the grassroots dimension of drugs policy in Iran.^5^ Ultimately, what governs the lives of precarious addicts is not the state’s imposition of order through disciplinary mechanisms or Islamising principles. Instead, it is an assemblage of public and private means aimed at *maintaining disorder*.^6^

## What is a ‘camp’ in Iran?

Known formally as short-term and medium-term in-patient treatment centres, these places are popularly known as *camps*. The word, rather than recalling the heinous reference to the Nazi concentration camps, refers to the expression *camp*-*e tabestani*, meaning ‘summer camps’ or ‘holiday camps’, that had become very much à *la mode* among middle-class Iranians in the 1990s.^7^ When the camps soon became publicly known for their dehumanising conditions, the label ‘camp’ proved that destiny may sometimes be in one’s name. As the Latin speakers would have said, *nomen omen*: ‘name is destiny’.^8^

Adapted after the philosophy of recovery of Narcotics Anonymous (NA), the equivalent of Alcoholics Anonymous for drugs, the camps are based on a detoxification process, usually lasting for one 21- to 28-day session.^9^ As charitable institutions, they are under the supervision of the Welfare Organisation – the state institution (formerly a ministry) charged with social assistance programmes – but they cultivate a close relationship with the police. In fact, the private rehab camps are regularly contacted by the police in order ‘to accommodate’ arrested drug users for rehab programmes, whenever the state-run compulsory camps are overwhelmed.^10^ While people referred by the police to the compulsory camps are treated free of charge, those referred to the rehab camps are expected to pay the fees, at least partially. In some cases, the Welfare Organisation covers the cost of in-patient treatment (but the bureaucratic process is time consuming, and its outcome uncertain). The camp owners admit that in no case have they demanded the full amount. They accept any monetary contribution the addict, or his family, is capable of making. Most of the time, however, people referred to private camps by the police refuse to pay and, consequently, as a camp owner explained me, ‘addicts are arrested by the police on Monday, and released by us [the camp owners] on Tuesday, because they don’t have money [to pay the fees]’.^11^ This has triggered criticism of the police, especially in view of the 2010 law reform that puts emphasis on ‘the judicial supervision of the arrest, treatment and release process’, which would require a judicial dossier to be opened for every referral. The camps are not legally required to keep the drug users against their will, so in the case of escape they do not take further action. Plus, there are no guards or police at their gates. The conservative newspaper *Keyhan* reminded the police that ‘the [private] camps have no right to maintain the addicts without a ruling of the judiciary; similarly they cannot let the addict leave the camp without approval of the judicial authorities’.^12^ Both practices are the rule rather than the exception. The dossier, alas, is missing.

With a drug-using population which has been described as among the largest worldwide, counting an estimated one and a half million out of a population of 80 million, the place of drugs is central to Iranian politics.^13^ Rooted in the cultural practice of people (ie opium use), Iranians have also acquired a new taste for drugs as exemplified by the popularity of heroin and methamphetamines (*shisheh*). More importantly, governance of the drug question has resulted in the adoption of progressive, forward-looking policies, starting from the early 2000s, despite the Islamic Republic’s uncompromising pledge to the War on Drugs. By 2005, the reformist government of Mohammad Khatami had introduced, under the pressure of an expanding HIV epidemic in prisons, a comprehensive set of ‘harm reduction’ measures. These included policies that remain often controversial in Europe and North America, such as needle exchange and distribution programmes, including in prisons (up to 2009), methadone maintenance and legitimisation of rehabilitation throughout the country.^14^ By 2017, two safe injection rooms for drug users operated in Tehran (nearby the opening scene’ parks) as part of a pilot project aimed at gathering evidence of this alternative model of drugs policy. This has put Iran ahead of most countries in the world in terms of drugs policy experimentation, despite a lack of reference to the Iranian case in international policy circles.

**Figure 1.**
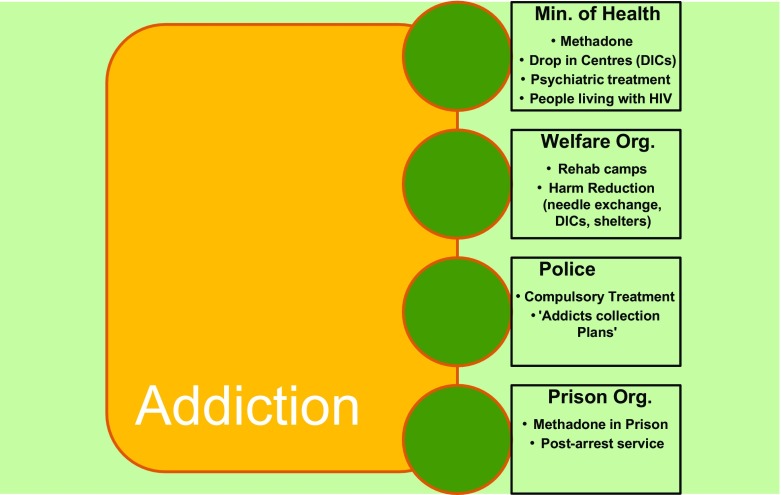
State institutions and addiction.

The institutions involved in the management of drugs policy are many: the Ministry of Public Heath oversees the work of methadone clinics; the Welfare Organisation is in charge of supervising the rehabilitation centres for drug addiction; and the police (*Niru-ye Entezami-ye Jomhuri-ye Eslami*, The Law Enforcement of the Islamic Republic, NAJA). Iran’s law enforcement, besides their duty of countering drug dealing, have also been involved in compulsory treatment programmes for drug addiction. All these institutions partake in the governance of Iran’s umbrella organisation for illicit drugs, the Drug Control Headquarters (DCHQ), which operates under the mandate of the president of the republic (Figure 1).

The coexistence of multiple visions of the drug phenomenon among these institutions has produced a contradictory set of responses. This ambiguity is enshrined in the legal framework of the drugs laws, which, in light of the emergence of crystal meth, were reformed in 2010.

## The oxymoronic laws

The 2010 drug laws reform, approved after complex negotiations within the Council for the Discernment of the Expediency of the State (aka Expediency Council),^15^ displayed the political and legal situation of drug (ab)use, following the approval of the harm reduction policy. Under the populist presidency of Mahmud Ahmadinejad (2005–2013), observers expected a setback for the harm reduction system. However, practices of support to drug (ab)users continued and effectively widened their quantitative scope after 2005. By 2007, there were 51 government facilities, 457 private outpatient centres and an additional 26 transition centres.^16^ By 2009, there were already 1569 treatment centres, 337 government centres and 1232 non-government centres operational throughout the country, providing services to 643,516 persons.^17^ Why and how this occurred is a question that goes beyond the scope of this article, but, in summary, one can tell that the expansion of public health measures under Ahmadinejad followed the logic of privatisation, which remained a driving principle during his mandate in all socioeconomic fields. In addition to this, one should mention the influence that the medical community and the NGOs had acquired over the late 2000s. Both contributed to the legitimisation of this approach during the post-reformist period. The fact that harm reduction and treatment of drug abuse had been included in the text of the General Policies of the Islamic Republic of Iran, emanating from the Expediency Council and approved directly by the Supreme Jurist Ali Khamenei, surely contributed to this process.^18^

The 2010 law reform materialised also the idiosyncrasies of the politics of drugs in the twenty-first century. The law itself provides a localised example of the paradigm of government with regard to the crises of drug (ab)use that the post-reformist government had faced. Firstly, the 2010-reformed law legitimised harm reduction practices that had been practiced since the early 2000s, by including them into an institutional order. Secondly, the law instituted specific centres for the implementation of harm reduction and rehab centres; these include state-run centres and private clinics as well as charitable and grassroots organisations. More crucially, the 2010 law established a distinction between those drug (ab)users who are willing to seek treatment and, indeed, to refer to a recognised institution (eg clinic, camp), and those who do not seek treatment, who therefore can be subjected to arrest. It also introduced the death penalty for people carrying more than 30 g of amphetamine-type stimulants (ATS). The line that divides the status of the categories remained thin and highly ambiguous. The reform produced an oxymoronic law.

**Figure 2.**
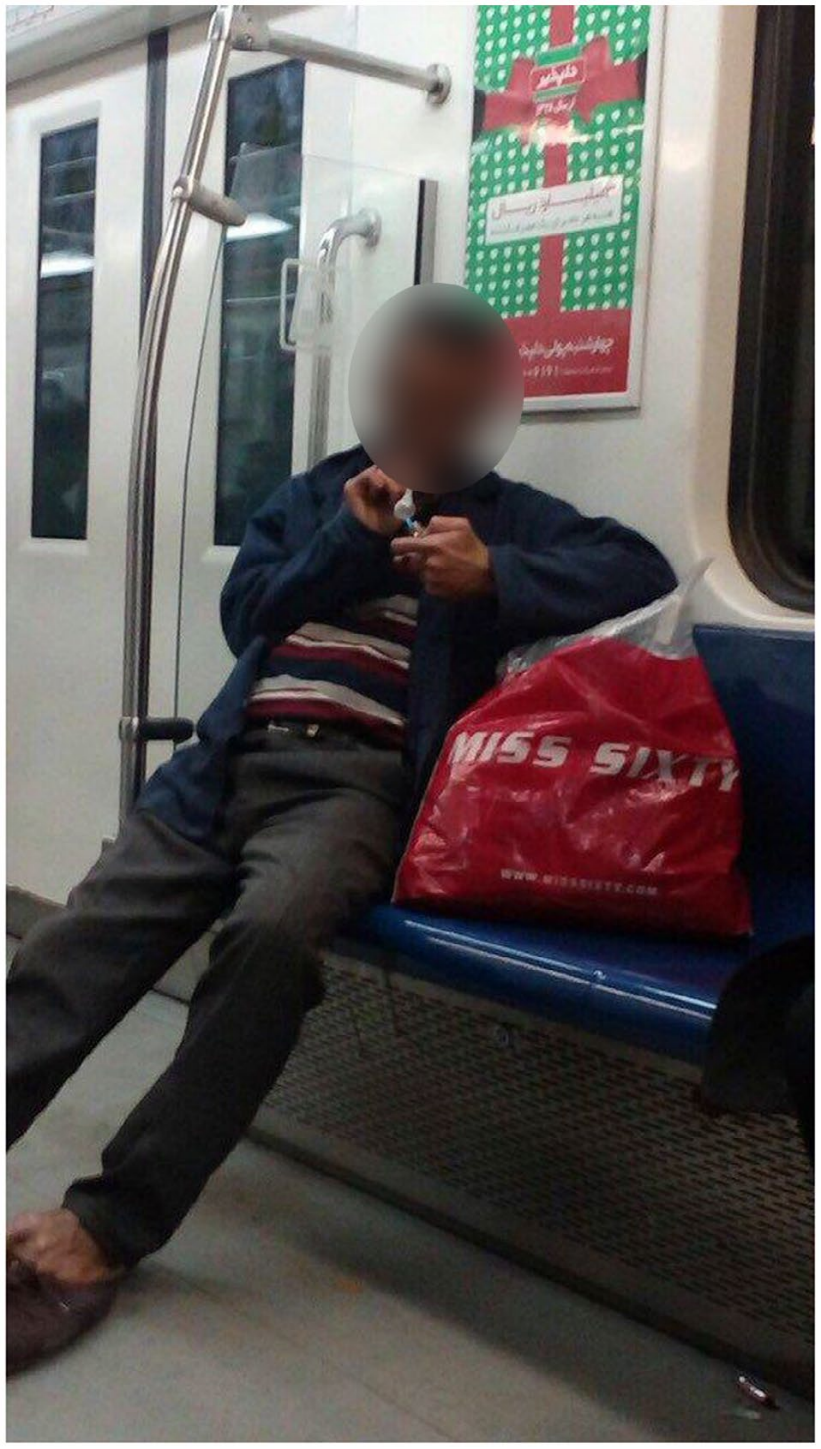
Meanwhile on the Tehran Metro: a shisheh smoker.

Note: This photograph circulated widely in September 2015 among Telegram App users. I received it in the drugs policy group (which includes leading members of the drugs policy community) ‘The Challenges of Addiction’.

The provisions of the 2010 law seemed to respond, in fact, to the necessities materialised by the expanding crisis of methamphetamine that had surfaced in public spaces. Public officials during the late 2000s agreed that people addicted to meth could not be cured, or that a cure for them was either unavailable or too expensive to be provided on a large scale.^19^ Meth users tend to be more mobile compared to people using heroin, who tend to ‘chill’ in a quiet place in contemplation of the spell of time. Iranian meth users had therefore a visible presence in the city, which caused concern among the law and order cadres as well as the public (Figure 2).^20^

This persuasion may have convinced the cadres of the state to seek mechanisms of intervention that were not necessarily coherent with each other, but which, from a state perspective, responded to the imperatives of public order. Pharmacological substitution programmes (methadone) and harm reduction practices (needle exchange) were inadequate to respond to the treatment of meth users. The medical community had expressed its impotency regarding the wave of meth use over the early 2010s, a fact that had implications also for the way the police needed to counter the issue. A lack of medicalised solutions implied that the practice of isolation and confinement became the primary response. This time, however, it was not through incarceration in state prisons, as had been the case since 1979, but through the work of different agents: state-run compulsory centres, private rehab and informal treatment camps.

## Humanitarian security

Since the implementation of the 2010 reform, the state had regularly intervened to collect street addicts and had confined them to compulsory camps, much to the astonishment of those who had worked towards the legitimation of harm reduction.^21^ The category of people targeted by these operations are the unemployed proletariat, a wageless class of mendicants, petty robbers, petty dealers, garbage collectors and ex-prisoners who fall outside the moral order of modern society and its political economy.^22^ In reality, part of the medical community and NGO sector had supported the text of the 2010 law on the basis that it legitimised harm reduction and proceeded towards a decriminalisation of addiction. The compulsory treatment camps, supporters of the 2010 law argued, were the necessary venue to medicalise addiction among those categories of (ab)users who could not be persuaded to seek treatment. It would be, they added, the safest way to introduce the addict into the cycle of treatment, thus facilitating his/her recovery.^23^ Yet the state-run camps often exposed situations of degradation, which prompted several officials to express publicly their opposition to this model, on the grounds that it neither brought results nor offered humanitarian support.

The origins of this institutional model can be traced back to the early years of the Ahmadinejad government. In 2007, already, the new head of the Drug Control Headquarters, Commander-in-Chief Ahmad-Moghaddam, announced that ‘the addict must be considered a *patient*-*criminal* who, if he is not under treatment, the court will rule for him compulsory treatment and the police will be the executor of a police-based treatment’. He then added, ‘we have to build *maintenance* camps; the police has already built camps for the homeless addicts and vagrants, which in the opinion of treatment officials can be used as maintenance camps for addicts for a certain period (emphasis added)’.^24^ This announcement can be considered an *ante tempore* elucidation of the 2010 law model. The continuity among homelessness, vagrancy and addiction is exemplified in the circumstances preceding Tehran’s Non-Alignment Summit in summer 2012. Ahead of the official visit of 120 state representatives, the police rounded up several thousand homeless people through the ‘addict collection programme’.^25^ Regardless of whether they were also drug users, the status of homelessness triggered the intervention of the police in cleansing the public space.

The fact that, genealogically, the compulsory treatment camps were formerly camps for the internment of vagrants and homeless people revealed the primary concern of the state with regard to the management of the public (dis)order and the lumpen classes of the cities.^26^ Much like in the 1980s, the officials adopted a language that emphasised the need to ‘quarantine’ problematic drug (ab)users, which had previously been addressed to the street vagrants,^27^ Yet this rhetoric did not anticipate a return to past forms of intervention; the post-reformist ‘quarantine’, instead, envisaged the presence and ‘supervision of doctors, psychologists, psychiatrists and infection experts as well as social workers’ and the referral, after the period of mandatory treatment, *of maintenance*, to ‘the non-state sector, NGOs and treatment camps’.^28^ This was largely discussed and never fully implemented. The rationale, it was argued, was to introduce ‘dangerous addicts’ and risky groups into the cycle of treatment, the first of which were managed by the state, through the ‘therapeutic police’, the law enforcement in charge of addiction treatment in the state-run camps.

After the approval of the 2010 law, large budgetary allocations were made by the government to the police in furtherance to the construction of compulsory camps. In 2011, ca. USD 8 million was allocated to the Ministry of Interior,^29^ with the objective to build a major compulsory treatment camp in Fashapuyieh, in the southern area of the capital Tehran. This first camp was designed to intern in the first phase around 4000 addicts (with no clear criteria of inclusion), with the number going up to 40,000 when the entire camp had been completed.^30^ Other camps were expected to operate in Iran’s major regions, including Khorasan, Markazi, Fars and Mazandaran.^31^ The deputy director of the DCHQ, Tah Taheri, announced that ‘about 250,000 people needed to be sent to the compulsory treatment camps by the end of the year’ as part of the governmental effort to curb the new dynamics of addiction.^32^ The ambitious plan had the objective, among other things, of unburdening the Prison Organisation from the mounting number of drug offenders, a move likely to benefit also the finances of the judiciary and the police, always overwhelmed by drug dossiers.

Yet the nature of the compulsory treatment camps resembles that of the prisons. After all, Iran’s drug legislation included a provision that required the separation of drug-related criminals from the rest of the prison population.^33^ This plan, which had been given support over the years, had never materialised on a large scale, leaving the prisons filled with drug offenders.^34^ By 2010, the prison population in Iran had increased to 250,000 inmates,^35^ equivalent to ca. 312 prisoners for every 100,000 people. The rate of incarceration remained significantly lower than that of the US and Russia, the top incarcerators worldwide, which counted 716 inmates per 100,000 and 415 per 100,000, respectively.^36^ But given that drug offences constituted the prime cause of incarceration in Iran, around 50 to 70%,^37^ the ratio speaks of the centrality of incarceration within the national drugs strategy.

Mostafa Purmohammadi, a prominent Iranian prosecutor,^38^ identified ‘addicted prisoners’ as one of the main concerns of the national prisons, and advised that the country needed to implement the mandatory treatment camps in order to alleviate the dangers and troubles of the prison system.^39^ Consequently, for the first time in many decades, Iran’s prison population had decreased by some 40,000 people in 2012, reaching the still-cumbersome number of 210,000 inmates. This datum, heralded as evidence of success by the post-reformist government, could be actually traced back to the introduction, on a massive scale, of the compulsory camps for drug addicts. In light of this consideration, the population confined in state institutions for charges of criminal behaviour (including public addiction) had actually mounted to almost double the number in prisons prior to 2010s.

Since the establishment of the Islamic Republic, the overall number of prisoners had multiplied by six times, and the number of those incarcerated for drug-related charges by 14 times, with one in three court cases allegedly being drug related in 2009.^40^ If during the reformist period the introduction of harm reduction had been prompted, among other things, from the HIV epidemic in prisons,^41^ the post-reformist government reacted with public outrage against the waste of money that the incarceration of drug addicts represented. In 2010, an official from the Prison Organisation outlined that maintenance costs were ca. US$1 per day per person, equivalent to 1.5 million per day, *ca.* 0.058% of the national budget (2009/2010).^42^ Researchers from state institutions demonstrated that treating drug addicts would cost an average of 15 times less than incarcerating them.^43^ In view of the number of drug (ab)users in prison, the creation of the compulsory treatment camps provided an alternative device for the maintenance and management of this population. The head of the judiciary, Ayatollah Sadegh Ardeshir Amoli Larijani, echoed these results, asking for a swift re-settling of addicted prisoners in the compulsory camps for treatment, which, instead of being under the supervision of the Prison Organisation, are managed by the DCHQ.^44^

At the same time, the government proceeded towards a significant expansion of methadone treatment, bringing treatment to more than 40,000 prisoners by 2014. Methadone, in this regard, represented an acceptable solution, as it was produced and controlled by the state, it was readily available through private and public clinics, and it greatly facilitated – by virtue of its pharmacological effects – the management of unruly subjects, such as drug addicts, in the contexts of prisons. Inspired by the relative success of these methadone programmes (in prisons, as much as outside), methadone treatment programmes were introduced inside some of the compulsory treatment camps supervised by the police. This, it seems, was identified as a productive way to introduce the highest numbers of drug abusers into the cycle of treatment, via allegedly less harmful drugs such as methadone. By familiarising arrested drug (ab)users with methadone, and by referring them to public methadone clinics, the authorities sought *to keep them off* more dangerous drugs, such as heroin.

Compulsory camps have also been part of the political economy of addiction in the Islamic Republic. By collecting, on a regular basis, street addicts from across the cities’ hotspots, especially in the capital Tehran, the police benefit from a substantial financial flow, justified by the expenses that it putatively incurs in managing the camps. Given that most of the state-run camps are known for their Spartan and down-to-earth conditions and services, it is implied that considerable amounts of money are filling the coffers of the police. This also implies that the police has a stake in the continuation of the activities of the compulsory camps and in the seasonal intervention aimed at gathering so-called ‘dangerous drug addicts’. The rationale behind this system is similar to that behind the instrumental arrests by the French police of ‘illegal migrants’ and ‘potheads’, or in the bonus/arrest cycle governing local policing in the US.^45^ Similar procedures operate in these different contexts, where the public presence of drug users justifies budgetary expansion for the police, out of the politics of police arrest numbers.

The camps embody a new mode of law enforcement, one which, instead of contesting public health interventions (eg harm reduction), uses its rhetoric and for reasons that are not ultimately humanitarian. It is a form of humanitarian security, or, using Didier Fassin’s oxymoron, ‘compassionate repression’.^46^ This strategy emphasised a management and maintenance of disorderly populations through coercive mechanisms, while leaving the larger group of drug (ab)users unbothered. But only a tiny portion of social disorder is targeted through the compulsory camps and the police.

## ‘I can check on the girls when I am not here!’

The mechanisms of intervention in the field of addiction have been more multifaceted than that of state-run camps. In fact, state-run camps entered this field alongside societal organisations that had set foot in the period preceding 2010. Rehabilitation centres have been operating legally or informally since the mid-1980s, although their extraordinary expansion can be traced back to the early 2000s and the new politico-medical atmosphere brought in by the reformists.^47^ Despite the promise of monetary subsidies from the state, most of the camps exist within an economy of subsistence based on donations from local communities, recovered addicts and mosques, and government cheques.

Official statistics reported in newspapers in the last decade reveal that one in 10 addicts in Iran is female.^48^ Yet there are also strong indications that a growing number of women are using crystal meth, which would logically imply that the percentage of female users has increased in the last decade. Women represent only 5% of all referrals to state institutions providing service for drug abuse, but a much higher presence is revealed in formal and informal treatment camps.^49^ The stigma for women is also more pervasive and, in several cases, female treatment camps have been set on fire because these camps were deemed immoral and ‘nest[s] of sexual vice’.^50^ Most of these places operate at the margins of the city, or inside apartments in popular neighbourhoods, in order to avoid being recognised as camps. No outdoor indication or explicit address is provided and the referrals occur through the state line of enquiry – ie the police – or through informal connections, via the family. Thence, the female treatment camps operate along those margins in which state intervention is rendered more problematic by the sensibility of gender issues, while popular resentment and stigma against them menaces their public presence. The state, for that matter, is reticent to allocate sufficient licences for the female camps, out of concern that the mushrooming of these institutions – once formally recognised by the state – would stipulate a less ambiguous datum of female drug (ab)use, one which might refute the static officialdom to which the government has hitherto pledged. In this way, it also secures flexibility in its cooperation with civil society. This condition marks more explicitly female drug (ab)users, but it also affects the phenomenon of treatment as a whole.

In 2011, the government approved the construction of one compulsory treatment camp for female addicts, to be located in the Persian Gulf region of Hormozgan. The site would host multiple categories of ab(users) whose common feature is their relation to the street (and the moral order): runaway girls trapped in drug abuse, streetwalkers, sex workers, female mendicants, and petty drug dealers and users. All these categories blur into each other, at least if one *sees like a state*.^51^ The location itself indicated that the site of this camp had to be peripheral; south along the coast of Hormozgan, the camp would work half as a public exile and half as a refuge from the public gaze. Hormozgan itself, however, had historically been characterised by heavy drug (ab)use, including among women, a fact that perhaps further justified the location of the camp there. The particularity of this project was also its nature as a joint venture between the state and a private organisation expected to manage the centre, an exception both to the 2010 law and to the practice in other camps.^52^ Given the ethical challenges of running a state-run treatment camp for women, the authorities partly disengaged from its routine administration and partly took advantage of the existing expertise and activism of NGOs dedicated for precarious women’s affairs.^53^ Yet a single female camp, located at the very periphery of Iran, could not comply with the necessities dictated by growing meth use among women. This void had been already filled by the establishment of female treatment camps, managed by private individuals or charities. I shall refer to one of them in particular, to which I was given repeated access over the course of my ethnographic fieldwork in 2014: the women’s camp situated in the city of Arak.

Operating as a sister branch of a male camp, the female camp could hardly be described as a camp. In reality, it was an apartment inside a four-storey building in a formerly middle-class area (mostly inhabited by public employees), today referred to generally as *payin*-*shahr*, ‘downtown’ (in Persian, it indicates ‘a popular periphery’). The apartment has three rooms and a small kitchen, with a long corridor used by the girls as a lounge to watch satellite TV (which is formally banned in Iran). The director had a number of close-circuit TV (CCTV) cameras installed in the apartment and has access to the video on her laptop; she could control the three rooms of ‘the camp’ from the desk of her office, or when she was at home, via an online application to which the CCTV cameras are connected. ‘In this way’, she explained, ‘I can check on the girls when I am not here’. She argued that the camp is self-managed by the girls themselves, who cook, clean and take care of the daily management of the place. They have a friendly, intimate relationship, she held, and she would like the place to be as comfortable and welcoming as possible for them. The door at the entrance of the apartment, nonetheless, has to remain locked at all times when she is not in; ‘otherwise the girls might run away and might go back to use drugs’. When I asked her ‘What if a person inside the apartment feels sick or needs urgent help?’ she justified this by saying that she can be reached at any time via mobile phone and that she checks on them regularly via the CCTV. She also relied on one of the girl in particular, Samira, who helped her by doing the grocery shopping and checking on the other girls while the director is away. Samira had been in the camp for a year and a half, since she was referred there by the female prison organisation. She had spent time in prison on several occasions for meth possession, aggression, armed robbery and ‘moral crimes’ (a euphemism for alleged sex work). Whether institutionalisation in this private camp had produced positive effects on her life is hard to say; certainly, she and I had the perception that her existence was suspended and that, despite the fact that she had stopped using drugs, addiction was still very much present in her life. In a way, this was nothing extraordinary: ‘I do not smoke anymore’, like ‘I do not drink anymore’, is part of the experience of people suffering from addiction, of the eternally ‘recovering addict’.^54^

The fee for a 21-day period is ca. US$110, which is one third of Iranians’ average monthly salary (ca. US$470).^55^ The people coming to the camp, as I discovered, did not live in Arak but usually came from other cities, since they wanted to avoid being recognised by their communities. This small apartment in particular had two girls from Khorramabad, a Kurdish woman from Kermanshah and another from Khuzistan. Three of the girls were interned in the camp as part of a compulsory treatment programme and were sent to the camp by the police. Since there is just one compulsory treatment camp for women – located at ca. 120 km from Arak – the authorities rely on private camps to accommodate these women, in which case they also pay the fees for their treatment. Generally, the director explains, the women referred by the police are more problematic, some of whom manifest serious health issues, while others have several criminal charges pending in their dossiers. One of them confided to me that the doctor had diagnosed her with *skizofrenì* (‘schizophrenia’) and asked me whether it was curable or not. It is not rare for these camps to refuse to take people referred by the police, out of fear of health contagion or in order to preserve their reputation.

The maintenance of order in the camp can indeed be troublesome. In the past, one of the women assaulted the director and threatened her with a knife. She was able to react, get a hold of the situation, and beat the woman, who had threatened her, under her feet. The director was condemned by the judge for her violent behaviour towards women interned in the camp. The camp was closed down for few months, before obtaining another licence under her husband’s organisation, which, I came to discover, is also a rehab camp for male addicts.^56^ The camp guaranteed a venue for ‘treatment’ and ‘control’ of female (ab)users who would otherwise be imprisoned. This does not imply that there is a statist strategy of covert manipulation through these organisations. Instead, this and other rehab centres operated as rhizomes of the state, a form of ‘government at distance’ of the drug phenomenon – a management of disorder.^57^

## State of camouflage and subterfuge

It has become common knowledge – if not a joke! – that contemporary Iranian society offers a wide range of informal, illegal centres for the provision of services (eg retirement houses, pharmacies, education centres), and that despite the government’s repetitive calls for their closure, these enterprises continue a lucrative existence.^58^ But the sheer quantitative dimension of the illegal addiction camps – nine out 10 rehab camps – signifies that this category affects more largely and, perhaps, categorically the phenomenon itself. Indeed, one could say that legal treatment camps in Iran are marginalia within the page of treatment. The phenomenon of camps suggests that these institutions, regardless of their public/private, legal/illegal status, exist in a continuum. Together they constitute a primary means of intervention – or mode of government – of addiction. Already in 2007, the government warned against the mushrooming of illegal camps and gave an ultimatum of three months to all camp managers to register for a licence at the Welfare Organisation.^59^ The DCHQ announced that ‘by the end of the year, the problem of the camps will be solved’,^60^ yet in 2014, the number of these institutions was higher than ever, with a veritable burgeoning across the country.

In Tehran alone, there were more than 400 illegal camps,^61^ while in Isfahan, out of 300 camps, only 16 had a licence.^62^ In the city of Arak, where I conducted part of my fieldwork, there were about 50 illegal camps, located near villages or main routes, or in private houses.^63^ These camps provide the opportunity for treatment for people and their families whose economic possibilities are limited. With the burden of economic sanctions being trickled down to popular strata, treatment in these institutions represented a more affordable and down-to-earth solution. Given the rootedness of the illegal camps, public officials started to change their approach, describing the camps as ‘a positive sign, because it implies that many people in Iran seek treatment’.^64^ As the country’s treatment capacity could not exhaust the demand for treatment, the officials hold, the camps are instrumental in this endeavour, even when they operate illegally.^65^

In the management of addiction, however, their role bypasses the logic of treatment and service provision. One could define the illegal camps using a Persian idiomatic expression: ‘the hand that captures the snake [*dast*-*e mar*-*gir*]’. Exclusively legal, bureaucratic or administrative means are deemed, according to the post-reformist govern*mentality*, insufficient and ineffective. To ‘treat’ addiction, hence, the state exploits the extra-legal function of the camps in areas from which the state itself had progressively disengaged, or has dissimulated its presence. The workings of the illegal camps can be sketched as such. In a situation when someone acts violently and volatilely, usually under the influence of meth, the family of the subject usually opts for the intervention of the camp personnel. This is regarded as a preferable option to the intervention of the police. By calling the illegal camp, the family avoids criminal charges, which could produce incarceration and time-consuming lawsuits, all of which cause greater economic burden to the family itself. Similarly, the intervention of the camp ‘thugs’ guarantees a lower profile for the family than that of the police, which, especially in popular neighbourhoods, can cause rumours and reputation damage (aka *aberurizi*).^66^

The police, too, seem to support the illegal camp system and, at times, inform the personnel of the camps about the location of the complaint. In this way, the camps take on the duties of the police, with regard to drug (ab)use.^67^ A police officer confirmed this informally during a conversation:

I am really happy that these camps exist; if a family calls us, instead of sending a soldier or a policeman, we call one of the people from the camps. So, if someone gets beaten, that’s the camp people, which also means that, if someone has to beat someone else, it’s always the camp people [and not the police]. Instead of taking the addict here to the police station, where he might vomit, feel sick and make the entire place dirty, he goes to the camp. Instead of coming here to shout and beat up people, or to bring diseases, HIV, he goes there. I call the camp if someone calls me.^68^

The camps are, thus, an apparatus of management of social crises and maintenance of disorder in the guise of addiction. *De facto*, many of the illegal treatment camps operate as compulsory treatment camps, because those secluded in them, for periods varying between 21 days and one year, have been forced into the camps. They have been forced not by the police, but by their local communities – usually their family members. The police play the part of the observer or the informant; they inform the camps, on some occasions, of the location and situation of a complaint, but no formal undertaking is initiated.

Inside the camps – several personal stories disclose – the managers adopt ‘alternative techniques’ for the treatment of addiction, the most infamous ones being ‘beating-treatment’, ‘water-treatment’, ‘dog-treatment’ and ‘chain-treatment’.^69^ Although there is generally a propensity towards sensationalising these accounts, the horrid accounts from inside the camps are telling about the lack of humanitarian purpose behind the workings of the camps. As in the case of the state-run *Shafaq* camp, which became the focus of a scandal in the 2010s, the deaths of interned addicts is the public signature of the camps’ practice.^70^ In 2016, all women were dismissed from the Shafaq centre, amidst allegations of violence.^71^

The liability for the crime remains exclusively with the camp managers, as noted in the statement of the police officer mentioned above. Camp managers are punished severely for casualties within the illegal camps. The state authorities have resorted, according to Islamic law, to *qesas*, retributive justice (‘an eye for an eye’), envisaging the death penalty for the camp managers (specifically in case the family of the victim refuses to accept the ‘blood money’).^72^ Although there are no clear data on the rate of deaths within the illegal camps, the reports in the newspapers suggest that these events have not been only sporadic between 2012 and 2016.

Among street drug (ab)users, the narratives of some of these camps gained mythological dimensions and instil vivid fear, a sentiment that is somehow reminiscent of a persecution. In this way, the camps fulfil a double promise: on the one hand, they intervene along the problematic margins of Iranian society (its ‘uncivil’ society), through the creation of extra-legal, unaccountable and, in view of their quantitative dimension, omnipresent institutions. They reduce the work of the police, while receiving nothing in exchange from the state. On the other hand, the camps are managed by former drug users, whose place within normative society remains unsettled. They struggle to find employment in regular businesses, and their housing status remains uncertain, and they often rely on temporary family accommodation; the camp, hence, becomes the only stable unit within their life, functioning as both occupation and residence. The post-reformist government succeeded in its quest ‘to socialise the war on drugs’ and to mobilise, by other means, civil society for statist ends.^73^ It is not, however, a co-option of civil society, which remains autonomous and unburdened by governmental diktats. In fact, this mobilisation does not prop up, as a grand strategy, any macropolitical agenda. With the camps providing motivation and an ecosystem in which to find their place within society, the camp owners rehearse a system in which the phenomenon of drug (ab)use dissolves into the machinery of treatment. Former (ab)users are employed in them and, whether willingly or involuntarily, mistreat other ‘addicts’, along the lines of previous securitising policies against drug users. This phenomenon is a form of ‘grassroots authoritarianism’,^74^ whereby social elements belonging to societal milieux of diverse nature partake in mechanisms of control, discipline and treatment fundamentally *maintaining disorder*. The camps’ relationship with the state remains ambiguous, based on rhetorical condemnation by public officials, haphazard prosecution by the judiciary, and clandestine connections, for instance in the referral of complaints by the police to the illegal camps.

The multitude of illegal treatment camps hints at another statist rationale. Licences for these camps can be provided by the state through the Welfare Organisation; but in order to do so, the government needs to guarantee minimum financial support, which given the large number of these centres would drain the budget from other treatment programmes, notably the compulsory treatment camps. ‘The closure of the illegal treatment camps is not part of the main policy of the state’, declared a public official in a conference, adding that ‘the existence of these camps is better than their non-existence, because their closure would mean disorder among the dangerous addicts’.^75^ In view of their indirect connection to the police, which sees them as a useful complement vis-à-vis problematic users, these institutions can be interpreted as part of the state effect. Despite their private and unrecognised status, they perform a public, state-sanctioned role.^76^

Despite almost a decade of reiterated calls to close the doors of the illegal treatment camps, these institutions maintain solid roots and operate, *qua* rhizomes, across the margins of rural and urban Iran. Their ubiquity has given rise to the phenomenon of *kamp*-*gardi*, ‘camp-touring’, which refers to the unending journey of the addict from camp to camp, a circumnavigation that rarely has a way out and often leads to the destitution of the individual, or to their incorporation in the activities of a particular camp.^77^ Those whose experiences have been more telling are often called by the rest of the community of the camps the ‘Marco Polo’, because they have visited as many camps as the Venetian traveller had done during his travels of the *Milione*.

**Figure 3.**
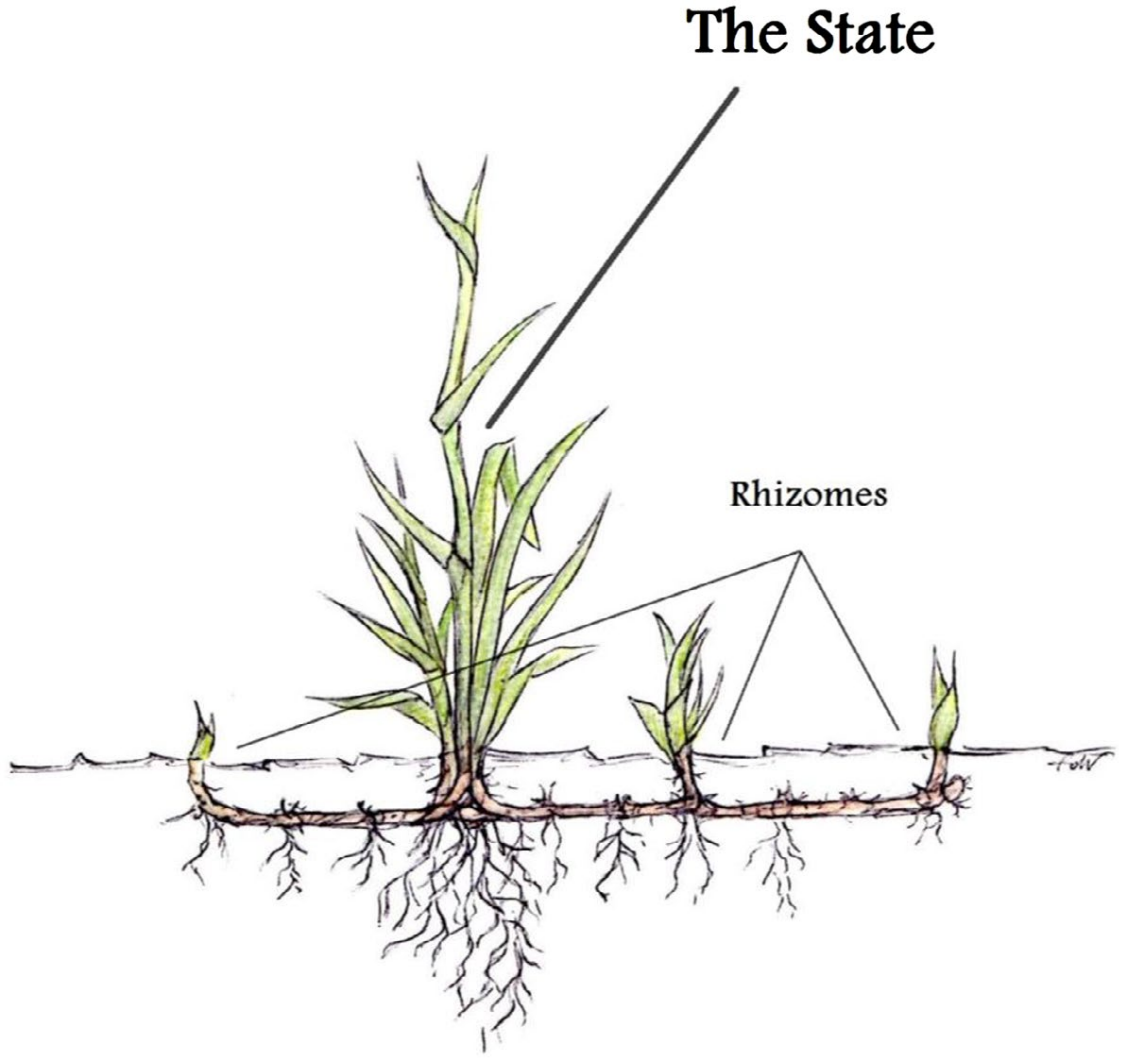
The state and its rhizomes.

Note: Drawing courtesy of Italian artist *Fruk* (aka Federica Di Violante).

There is no spelled-out policy about the role of illicit rehab camps, but the daily routines of drugs policy reproduce what works as a maintenance assemblage. It is at the level of micropolitics that this condition is best captured, as illustrated above. The camps operate as a rhizome of the state, which instead of reproducing vertical lines of control and discipline, becomes diffused and horizontal – similar to the grassroots (rhizomes) of a tree. When societal control is practiced, this is cropped out through the rhizomes that stem from the horizontal roots of the state itself, camouflaged as other forms of intervention, the illegal world of treatment dealing with the illegal life of drugs users. This forms what I define as *maintaining disorder* (Figure 3).^78^

## Conclusions: maintaining disorder

There is no fundamental rupture, or watershed, among the state-run compulsory camps, the informal, illegal camps and the Harandi Park model (Table 1). Together they fulfil an ultimately political objective in reaction to a phenomenon that has permanently been framed as a crisis. Because of that, the rehab camps enter a field of interest to the state – one could say an expediency – in which the underlying rule is the *maintenance of disorder*, the management of a permanent crisis, an ordinary emergency embodied in the presence of drugs users. It is not, as one would expect in the Islamic Republic, a matter of moral evaluation, religious justification or variation in (post-)Islamist change. Islam and theological considerations do not have a place in this matter and, in fact, found no place in the narratives described in this article.

**Table 1.** Rehabilitation ‘camps’.

	State run	Private	Illegal
Legal status	Legislated under Article 16 of the 2010 drug law.	Legislated under Article 15 of the 2010 drug law.	Illegal.
Management	Managed by the NAJA, with support from the Welfare Organisation, Ministry of Health.	Managed by private organisations, charities, associations, etc.	Managed by private individuals, or group of people.
Funding	Receive direct state funding, through DCHQ.	No direct funding from the state. Fees are applied for treatment periods of ca. 21 days. Donations from families. Subsidies from Welfare Organisation per treated addict.	No subsidies or governmental funding. Fees apply per person. Donations from local communities. Negotiations for poor families.
Personnel	Social workers, police officers, medical professionals (on paper). In practice, police and local aides.	Former drug users; NA members; social workers and volunteers.	Former and current users.
Methods	Detoxification; in some facilities, methadone substitution is provided. *Narcotics Anonymous* (NA) support potentially available.	Detoxification, mostly based on NA 12 steps; some organisations adopt specific therapies, eg music therapy, meditation.	Detoxification, also through violent means and coercion.
Target group	Street drug users; homeless drug users; *Patoqs*. Polydrug users.	Depends on the organisation; mostly, lowermiddle-class drug users, both urban and rural. In specific cases, upper-class people.	Poor drug users, young people, men under psychotic attacks; mostly *shisheh* and polydrug users.
Means of referral	Arrests. Police operations, drug addicts’ round-up plans. Coercive.	Voluntary referral, through advertisement, word of mouth.	Family, community referral; police referral. Mostly coercive.
Fees	Free.	Set fees (government decree); often negotiated.	Flexible fees, based on status, negotiation.

There are other social fields where what the article demonstrated for drugs can be similarly ascertained. Here are a few cursory examples: Iranian authorities, based on religious interpretation, allow and actively sponsor so-called ‘temporary marriages’ (*sigheh* in Farsi), while *de jure* punishing premarital sex. Temporary marriage is a contractual agreement (as all marriage is according to Islamic jurisprudence) in which the two parties determine beforehand the duration of the marital bond. In practice, temporary marriage has resulted in the tolerance of sex work, especially in sites of religious pilgrimage (Qom, Mashhad), but has also become an expedient for people not willing to engage in a permanent union.^79^

Similarly, since the late 1980s, the authorities have legislated in favour of gender reassignment surgery (sex change), legalising and providing welfare support for people who want to change gender, while denying legal status to homosexuals.^80^ This has gained the Islamic Republic a reputation as a leading centre for sex change surgery worldwide.

In its ethical dilemma and political incongruence, the drugs and addiction question resembles the above-mentioned cases, for the Islamic Republic has systematically criminalised drug offenders and punished them with draconian measures, while it has also provided one of the most progressive and controversial sets of public health programmes for drug (ab)users. In quantitative scope, however, the drugs case is far more conspicuous and mainstream than the issues of sex work and sex change. As seen in the narratives of the article, maintaining disorder, instead of imposing order, had governed the logics of public interventions on drugs. This approach prompts limited tolerance of public drugs use – and the life of ‘addicts’ – in the case of Harandi Park as well as in the existence of illegal treatment camps, where their life is subject to informal control. The contradictions and articulations of this assemblage ultimately show the primacy of political prerogatives over ideological lineages.

This frame of analysis may prove useful for the understanding of political processes at large: in the tolerance of opposition and dissent within ambiguous limits of institutional politics; in the dynamics of clerical opposition within the Islamic Republic; and in the enforcement of public codes of conduct, as related to the *hijab*. In this article, maintaining disorder defined the governmental approach to the drug crisis but also the state’s ideology of practice. This art operates at the level of fabrication, make-believe and execution, confuting the notional existence of law and the state, as seen in the case of the camps and the park. In intervening on the phenomenon of drug (ab)use, the Islamic Republic envisioned its modus operandi as one based on secular pillars of management. The result has been a paradigm of government that deals with social crisis and ethical disorder without the objective of solving it, or of imposing a strict script on it. Instead, what is distinctive is the engendering grey area of state control/repression/compassion in dealing with precarious lives. This grey area situates Iran’s case in the global grey areas of the immigration detention centres, the homeless shelters, the terrorist detainees’ camps. Seen this perspective, maintaining disorder is not specific to Iran, but is a paradigm of government in the current epoch.

## Funding

This work was supported by the Wellcome Trust Society & Ethics Doctoral Scholarship [Grant No. WT101988MA]. The symposium where the paper was first presented was made possible by Wellcome Trust Small Grants [Grant No. 202095/Z/16/Z].

## Note on Contributor

***Maziyar Ghiabi*** is currently a Visiting Researcher at CAMES, American University of Beirut. In February 2017 he successfully passed his DPhil in *Politics* at the Department of Politics and International Relations, University of Oxford. Between 2013 and 2017 he was a Wellcome Trust Scholar in Society & Ethics with a research project on the history and politics of drug addiction in modern Iran. From September 2017, he takes up his new post as a Postodoctoral Fellow at the École des hautes études en sciences sociales (EHESS) in Paris, with a project on historical and political ethnography in the context of drug violence. Maziyar has also published on political ethnography of revolt and civil war across the Mediterranean. He served as Guest Editor to the *Third World Quarterly* Special Issue on ‘Drugs, Politics and Society in the Global South’.

## Disclosure statement

No potential conflict of interest was reported by the author.

## Notes

1. A Persian proverb equivalent to ‘Don’t count your chickens before they hatch’.

2. Accounts of Harandi Parks also appeared in newspapers. See *Iran*, October 5, 2015.

3. This model could potentially develop into something similar to Sao Paulo’s *De Braços Abertos* project in *Crackolandia*; see Prefertura da Cidade de São Paulo, ‘O Programa do Braços Abertos’, available at https://www.prefeitura.sp.gov.br/cidade/secretarias/upload/saude/DBAAGO2015.pdf

4. Deleuze, *Due Regimi di Folli e Altri Scritti*, 3.

5. See Garcia, *Pastoral Clinic*; Zigon, *HIV Is God’s Blessing*; Raikhel and Garriott, *Addiction Trajectories*.

6. Similarly, a police officer during the Great 8 (G8) in Genoa in 2001 asserted that the government did not want the police to establish order, but *to maintain the disorder*. Reported by Agamben, “Comment l’Obsession Sécuritaire Fait Muter.”

7. I am grateful to Fariba Adelkhah for pointing out this aspect.

8. This is different from the French case described by Fassin, where activists as well as Sarkozy himself who opposed these places used the word ‘camp’ instead of ‘centre’ to delegitimise them. In Fassin, *Humanitarian Reason*, 133–4.

9. Rehabilitation in these centres follows the 12-step process established by NA. Most of the camp managers and assistants are also members of NA and the camps often have logos and banners indicating their affiliation to the NA superstructure. Based on ethnographic observations in rehab camps in several Iranian cities, 2012–2016.

10. Interview with the manager of a camp in the village around Arak, 2 April 2014.

11. Interview in Hasanabad, April 3, 2014.

12.
*Keyhan*, June 10, 2012.

13. This is the official government data and it has been stable over the last few decades, despite significant changes in the drug market and evidence of a rise in drugs use. There are other estimates, at times declared by government officials themselves, which refer to higher numbers, eg 2–5 million; see United Nations Office of Drugs and Crime (UNODC), “Drug Prevention Treatment and HIV AIDS,” https://www.unodc.org/islamicrepublicofiran/drug-prevention-treatment-and-hiv-aids.html

14. Ghiabi, “Drugs, Addiction and the State,” chapter 6.

15. All Iranian legislations are discussed in Parliament with the exception of drug laws, which are legislated exclusively in the Expediency Council.

16. Calabrese, “Iran’s War on Drugs.”

17. DCHQ, “Drug Control in 2009.”

18. The text of the General Policies can be found on the website of the Expediency Council, available at https://maslahat.ir/DocLib2/Approved%20Policies/Offered%20General%20Policies.aspx

19. Interview with Tahernokhost, Tehran, September 2012; and interview with Emran Razzaghi, Tehran, September 2012.

20. For instance, the increase of reckless driving and lunatic behaviour in public, described in journalistic pieces, hints at this. See *Aftab-e Yazd*, August 30, 2008. For stories about *shisheh*, which went viral, see *Mehr*, December 22, 2012, available at https://www.mehrnews.com/news/1770270/ ; and *Sharq*, November 3, 2013, available at https://sharghdaily.ir/1392/08/14/Files/PDF/13920814-1874-22-12.pdf

21. These operations are usually called *nejat*, ‘salvation’, and prior to 2010 they contemplated incarceration for short periods.

22. In the presidential election of May 2017, the issue of marginalisation became a central theme. Addiction was a key category of this debate; for a discussion of the category of ‘lumpen’, see Denning, “Wageless Life.” One could see how the addiction camps are the postmodern equivalent of the *lazzaretti* and *lazzaroni*, the leper hospital and leper.

23.
*Mehr*, September 26, 2012, available at https://www.mehrnews.com/news/1608510/

24.
*Iran*, May 12, 2007.

25. Ethnographic observation while working as a research intern at the United Nations Office on Drugs and Crime, September 2012. Accounts available also in *Tabnak*, September 12, 2012, available at https://www.tabnak.ir/fa/news/272411

26. A similarity that is reminiscent of the 1980s approach; see Ghiabi, “Drugs and Revolution in Iran.”

27.
*Hamshahri*, April 30, 2008.

28. Ibid.

29. After 2009, incidentally, the head of the DCHQ was Mostafa Najjar, then Interior Minister.

30.
*Jam*-*e Jam*, February 28, 2011, available at https://www1.jamejamonline.ir/papertext.aspx?newsnum=100836959206

31. Ibid.

32.
*Jam*-*e Jam*, May 16, 2011.

33. In Article 42 of the Reformed 2010 Drugs Law; the text is available at https://rc.majlis.ir/fa/law/show/99642

34.
*Salamat News*, May 8, 2012.

35.
*Tabnak*, February 8, 2013 available at https://www.tabnak.ir/fa/news/301709

36. International Center for Prison Studies, *World Prison Population List*, 2013, available at https://www.apcca.org/uploads/10th_Edition_2013.pdf

37.
*Eqtesad News*, February 11, 2017, available at https://www.eghtesadnews.com; It is said that up to 80% of death sentences are due to drug trafficking charges. See *Sharq*, August 8, 2010.

38. He was Minister of Interior between 2005 and 2008, and is the Minister of Justice in Rouhani’s current government.

39.
*Ruzegar*-*e Ma*, August 27, 2011.

40.
*Hamshahri*, January 22, 2009.

41. Behrouzan, “Epidemic of Meanings.”

42.
*Jam*-*e Jam*, April 11, 2010; The equivalent of ca. US$150,000 per day.

43.
*Hamshahri*, April 30, 2009.

44.
*Aftab News*, June 16, 2011, available at https://aftabnews.ir/prtb89b8wrhb5fp.uiur.html

45. For the Iranian case, see *Mardomsalari*, September 22, 2012; For an ethnographic study of French police strategies and governmentality, see Fassin, *La Force de l'Ordre*. A discussion of morality and the police instead can be found in Fassin, “Maintaining Order”.

46. Fassin, *Humanitarian Reason*, 135.

47. Christensen, *Drugs, Deviancy and Democracy*.

48. In a decade, the number of female drug ‘addicts’ has almost doubled, according to the DCHQ; see *Fararu*, August 2, 2016, available at https://fararu.com/fa/news/283802/

49.
*Hamshahri*, June 24, 2009.

50.
*Sharq*, July 24, 2012.

51. Scott, James C. *Seeing like a State.*

52.
*Kh*a*baronline*, June 10, 2011, available at https://www.khabaronline.ir/print/156388/

53. A good example is *Khaneh*-*ye Khorshid*, which had collaborated with the state in providing welfare services to sex workers in Tehran.

54. On this oxymoronic figure, see the ‘detoxified addicted’ in Deleuze, *Due Regimi di Folli e Altri Scritti*, 119.

55. Statistical Center of Iran, available at https://www.amar.org.ir

56. I later came to learn that this story was also widely reported in the news. See *Sharq*, September 24, 2012.

57. For a discussion of ‘government at distance’, see Hibou, *Privatizing the State*, 15.

58.
*Hamshahri*, May 4, 2008.

59.
*Hamshahri*, March 9, 2010. The Welfare Organisation has an ad hoc office for drug addiction, which issues these licences.

60. Ibid.

61. Ibid.

62. Interview with Hamid-Reza Tahernokhost, Drug Demand Reduction, UNODC, Tehran, September 2012.

63. Interview with Hasan Solhi, head of a state-run clinic in Arak, March 2014.

64.
*Jam*-*e Jam*, May 16, 2011.

65.
*Hamshahri*, January 25, 2010.

66. Ethnographic notes in popular neighbourhoods of Shush, Dowlatabad, in Tehran; and Futbal, Cheshm-e Mushak, in Arak. See also *Jam*-*e Jam*, April 16, 2012.

67. Interview with Tahernokhost, September 2012.

68. Interview with a former police officer in Arak, September 2014. There is a telling comparison with the work of the Red Cross in the French migration centre of Sangette (later the ‘Jungle’), where the aid workers are found policing the centre instead of the police who have an ‘almost benevolent attitude’. See Fassin, *Humanitarian Reason*, 139.

69.
*Andisheh*-*ye Nou*, October 12, 20009; *Salamat* News, October 22, 2013, available at https://www.salamatnews.com/news/85137/

70. For an overview, see *Tabnak*, December 25, 2013, available at https://www.tabnak.ir/fa/news/366881

71. The public diatribe can be read here: *Azad News Agency*, April 25, 2016, available at https://www.ana.ir/news/98756

72.
*Etemad*-*e Melli*, June 11, 2009.

73. Intervention by DCHQ official Hami Sarrami at the Addiction Studies Conference, Tehran, September 2015.

74. Shore, Wright, and Però, *Policy Worlds*, 114.

75.
*Jam*-*e Jam*, December 19, 2010.

76. Shore, Wright, and Però, *Policy Worlds*, 14, 114.

77. See also *Jam*-*e Jam*, December 19, 2010.

78. Navaro-Yashin, *Faces of the State*, 119 and 134.

79. Haeri, *Law of Desire.*

80. Najmabadi, *Professing Selves.* The cost of the entire process is covered by the Welfare Organisation.
